# Brain iron deposition in primary insomnia—An in vivo susceptibility‐weighted imaging study

**DOI:** 10.1002/brb3.1138

**Published:** 2018-12-11

**Authors:** Lin Chen, Xin Wei, Chen Liu, Chuanming Li, Zhenhua Zhou

**Affiliations:** ^1^ Department of Psychology Third Military Medical University (Army Medical University) Chongqing China; ^2^ Department of Radiology Southwest Hospital Third Military Medical University (Army Medical University) Chongqing China; ^3^ Department of Radiology The Second Affiliated Hospital of Chongqing Medical University Chongqing China; ^4^ Department of Neurology Southwest Hospital Third Military Medical University (Army Medical University) Chongqing China

**Keywords:** cognitive impairment, iron deposition, MRI, primary insomnia

## Abstract

**Background:**

To study the brain iron deposition and its relationships with cognitive impairment and sleep quality in primary insomnia (PI).

**Methods:**

Thirty‐five patients with PI and 35 volunteers underwent MRI scanning using high‐resolution susceptibility‐weighted imaging sequence. Bilateral anterior cingulate cortices, posterior cingulate cortex, hippocampus, caudate nucleus, globus pallidus, putamen, thalamus, red nucleus, substantia nigra, parietal cortex, and frontal white matter were selected as regions of interest. The phase shift values of the above areas were compared between the two groups. Partial correlations between phase shifts values and neuropsychological scale scores including Pittsburgh Sleep Quality Index, Insomnia Severity Index, Mini Mental State Examination (MMSE), Montreal Cognitive Assessment (MoCA), Activities of Daily Living Scale, and Clinical Dementia Rating of the PI patients were analyzed.

**Results:**

Compared with the normal controls, the PI patients showed significant lower MMSE and MoCA scores and increased phase shift values in the left caudate nucleus, left putamen, left hippocampus, and bilateral thalamus (*p *<* *0.05). Close correlation was found between the phase shift value of the left hippocampus and the MMSE scores of the PI patients (*R *=* *−0.447, *p *<* *0.01).

**Conclusion:**

The PI patients exhibited significant cognitive impairment and increased iron deposition in several brain regions. The iron concentration of the left hippocampus is a biomarker of cognitive impairment and may play an important role in the pathophysiological mechanism.

## 1 INTRODUCTION

Primary insomnia (PI) is a disorder of sleeplessness including poor sleep quality or short sleep time. It afflicts 10%–15% of the adult people in the world (Cheung, Bartlett, Armour, & Saini, 2013; Taylor, Lichstein, & Durrence, 2003). Patients with PI commonly have impaired daytime function and many physiological dysfunctions (Buysse et al., 2007; Liu et al., 2014; Riedel & Lichstein, 2000). Electroencephalogram studies have found that PI patients showed elevated spectral power values in the beta and sigma frequency band (Spiegelhalder et al., 2012). Greater global cerebral glucose metabolism during sleep was also found using positron emission tomography in PI (Nofzinger et al., 2004). As a noninvasive technique, resting‐state functional magnetic resonance imaging can detect spontaneous neural activity in the brain. Patients with insomnia exhibit decreased frontoparietal cortex activation during working memory task performance (Drummond et al., 2013; Li et al., 2014). However, the mechanism by which the PI influences the brain function of the specific brain regions has not been fully elucidated.

Iron contributes to many biological processes, including oxygen transport, protein expression regulation, and cell growth. Previously, it has been found that excessive brain iron deposition plays an important role in the arise and development of brain function disorders and cognitive impairment (Haller et al., 2010; Liu et al., 2015; Smith, Harris, Sayre, & Perry, 1997). Susceptibility‐weighted imaging (SWI), which is combined by the phase images and the magnitude images, is sensitive to the paramagnetic effect of iron particles (Haacke, Mittal, Wu, Neelavalli, & Cheng, 2009; Mittal, Wu, Neelavalli, & Haacke, 2009). It has been proved reliable to measure tissue iron concentration, which is consistent with the autopsy examination results. We hypothesized that (a) there is brain iron deposition in PI patients in multiple brain regions and (b) individual cortical brain iron deposition in specific regions could correlate with the brain function alterations of PI patients.

## 2 MATERIALS AND METHODS

### 2.1 Subjects

Thirty‐five patients with PI were recruited from the sleep disorder clinic of our hospital (Table 1). The diagnosis criteria were made according to the International Classification of Sleep Disorders and Diagnostic and Statistical Manual of Mental Disorders, version 4 (DSM‐IV). The Pittsburgh Sleep Quality Index (PSQI) was used to assess the sleep quality and disturbances. The Insomnia Severity Index (ISI) was used to evaluate subjective symptoms of insomnia. All patients underwent formal neuropsychological assessment using the following tests: Mini Mental State Examination (MMSE), Montreal Cognitive Assessment (MoCA), Clinical Dementia Rating (CDR), Activities of Daily Living Scale (ADL), Figural Recognition Test, Verbal and Categorical Fluency Test, Auditory Verbal Learning Test, Boston Naming Test, Hamilton Anxiety Scale, and Hamilton Depression Scale. Exclusion criteria were as follows: (a) the patient who had an abnormal brain in conventional CT or MRI; (b) the patient who had serious organic disease or severe mental disease. Thirty‐five age, gender, and education levels matched normal controls were recruited from the community. The exclusion criteria including: nervous system diseases such as brain trauma, tumor or hemorrhage, alcohol or drug abuse, systemic disease or other MRI contraindication. All subjects with severe depression (Hamilton Depression Rating Scale ≥18) or dementia (MMSE < 24) were also excluded. All of the participants were right‐handed. Written informed consent was signed by every participant. This study was conducted in accordance with the Declaration of Helsinki. All the procedures were approved by the Medical Ethics Committee of our institution.

**Table 1:** Demographic and clinical data of the primary insomnia patients and control groups

	PI patients (*n *=* *35)	Control subjects (*n *=* *35)	*p*‐value
Age (years)	43.2 ± 11.7	40.1 ± 6.4	>0.05**
Education (years)	10.5 ± 3.7	11.2 ± 4.3	>0.05**
Gender (male/female)	12/23	11/19	>0.05*
PSQI	12.9 ± 3.4	2.3 ± 0.8	<0.01**
MMSE	25.1 ± 1.4	29.0 ± 1.0	<0.05**
MoCA	24.7 ± 1.7	29.4 ± 0.9	<0.01**
HAMA	8.4 ± 3.2	1.7 ± 1.3	<0.01**
HAMD	7.8 ± 2.7	0.6 ± 0.5	<0.01**
ADL	100 ± 0	100 ± 0	

*Note*

Data were expressed as mean ± *SD*.

ADL, Activities of Daily Living Scale; HAMA, Hamilton Anxiety Scale; HAMS, Hamilton Depression Scale; MMSE, Mini Mental State Examination; MoCA, Montreal Cognitive Assessment; PSQI, Pittsburgh Sleep Quality Index.

*p ‐value was obtained using the two‐tailed chi‐squared test. **p ‐value was obtained using the two‐sample, two‐tailed *t* test.

### 2.2 Magnetic resonance imaging

All subjects were scanned on a 3.0 T whole body MRI scanner (Magnetom Trio, Siemens Healthcare, Erlangen, Germany). Susceptibility‐weighted images were obtained with the following parameters: TR/TE: 56/42, flip angle: 20°, section thickness: 2 mm, field of view: 23 × 17 cm, matrix size: 348 × 320. For SWI, postprocessing was performed according to the previous literature (Haacke, Xu, Cheng, & Reichenbach, 2004). The conventional sequences include the transverse T1‐weighted images (TR: 200 ma, TE: 2.78 ms, matrix: 384 × 384, flip angle: 70°, voxel size: 0.7 × 0.6 × 5 mm^3^) and fluid‐attenuated inversion recovery (FLAIR) images and susceptibility‐weighted images (TR: 9,000 ms, TE: 93 ms, TI: 2,500 ms, matrix: 256 × 256, flip angle: 130°, voxel size: 0.9 × 0.9 × 4 mm^3^).

**Figure 1:**
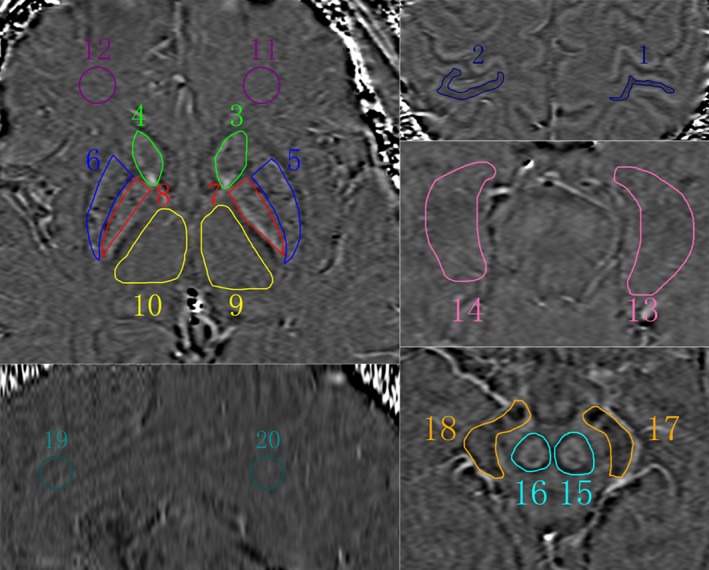
Region of interests in MR images. 1, 2: parietal cortex; 3, 4: caudate nucleus; 5, 6: putamen; 7, 8: globus pallidus; 9, 10: thalamus; 11, 12: frontal white matter; 13, 14: hippocampus; 15, 16: red nucleus; 17, 18: substantia nigra; 19: anterior cingulate cortex; 20: posterior cingulate

Images were analyzed by two independent radiologists who have 10 and 11 years of experience in MRI with SPIN software (Signal Processing in NMR, Version 1751, MRI Institute for Biomedical Research, Detroit, MI; http://www.wayne.edu/download.htm). SWI original images were first processed through a 32 × 32 high‐pass filter. Then, the high‐pass filtered images were weighted by the coil sensitivity factor and combined to a single complex image. Finally, the corrected phase images and normalized phase mask were created and multiplied with the magnitude image to produce the final SWI and phase images. Bilateral anterior cingulate cortex, posterior cingulate cortex, hippocampus, caudate nucleus, globus pallidus, putamen, thalamus, red nucleus, substantia nigra, parietal cortex, and frontal white matter were selected as region of interest (ROI) (Figure 1). The area of the ROI was 100 pixels in a circular shape. The Siemens Phase Unit was measured from each ROI and converted into radians using according to the previous literature (Haacke et al., 2007).

### 2.3 Statistical analysis

SPSS software (version 18.0; SPSS, Chicago, IL) was used for statistical analysis. Comparisons of phase shift values between the PI patients and the normal controls were performed using two‐sample *t* tests. Partial correlations between phase shifts values and the scores of MMSE, MoCA, ADL, PSQI, and ISI tests were analyzed for the PI group. Age, gender, and education levels were imported as covariates and the significance was set at *p *<* *0.05 corrected with Bonferroni correction. Intraobserver variability was analyzed by using intraclass correlation coefficients.

## 3 RESULTS

The PI patients and normal control volunteers had no significant difference in age, sex, and education years (*p *>* *0.05).

**Figure 2:**
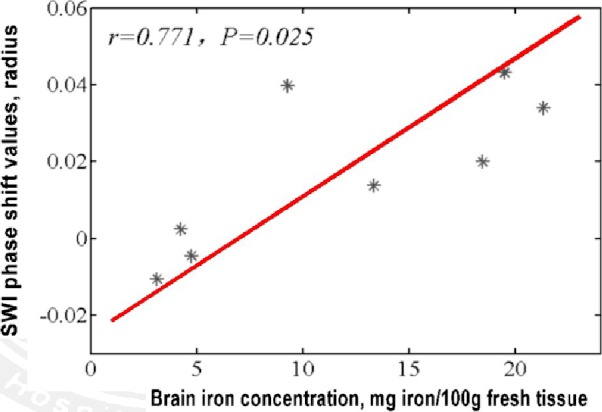
Correlation of the phase shift values of our normal controls with real brain iron concentrations of golden standard

The phase shift values in the putamen, caudate nucleus, globus pallidus, substantia nigra, red nucleus, thalamus, frontal white matter, and hippocampus of our normal controls were −0.0106, 0.0199, 0.0431, 0.0137, 0.0398, 0.0339, −0.0046, 0.0024, respectively. A close positive correlation (*R *=* *0.771, *p *<* *0.05) between the phase shift values of our normal controls and the regional real iron concentrations reported by the literatures (13.32, 9.28, 21.30, 18.64, 19.48, 4.76, 4.24, and 3.13 mg per 100 g of wet weight) was found (Figure 2).

**Figure 3:**
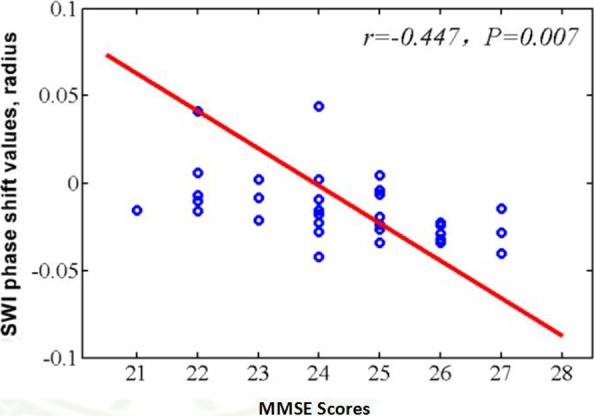
Correlation between the phase shift value in the left hippocampus and Mini Mental State Examination scores of the primary insomnia patients

Compared with normal controls, the PI patients have significantly lower MMSE, MoCA scores, and higher PSQI, HAMA, HAMD scores. The PI patients showed significant increased phase shift values in the left caudate nucleus, left putamen, left hippocampus, and bilateral thalamus (*p *<* *0.05; Table 2). Close correlation was found between the phase shift value of the left hippocampus and MMSE scores (*R *=* *−0.447, *p *<* *0.05; Figure 3).

**Table 2:** Regional phase shift values of healthy controls and primary insomnia patients (mean ± *SD*)

ROI	PI (*n *=* *35)	NC (*n *=* *35)	*t* value	*p*‐Value
Left PC	0.0011 ± 0.0111	−0.0016 ± 0.0096	0.903	0.370
Right PC	−0.0086 ± 0.0192	−0.0119 ± 0.0109	1.751	0.085
Left CN	0.0563 ± 0.0104	0.0387 ± 0.0174	5.013	0.000*
Right CN	0.0500 ± 0.0266	0.0409 ± 0.0185	1.576	0.120
Left PU	0.0450 ± 0.0156	0.0195 ± 0.0148	6.712	0.000*
Right PU	0.0079 ± 0.0087	0.0073 ± 0.0254	0.131	0.896
Left GP	0.0459 ± 0.0340	0.0357 ± 0.0196	1.452	0.151
Right GP	0.0313 ± 0.0280	0.0323 ± 0.0188	0.162	0.872
Left TH	0.0235 ± 0.0272	−0.0101 ± 0.0244	5.201	0.000*
Right TH	0.0143 ± 0.0139	0.0009 ± 0.0141	3.867	0.000*
Left FWM	0.0063 ± 0.0181	−0.0011 ± 0.0129	1.390	0.140
Right FWM	0.0083 ± 0.0153	0.0059 ± 0.0223	0.517	0.607
Left HP	−0.0151 ± 0.0191	−0.0220 ± 0.0225	2.473	0.016*
Right HP	0.0026 ± 0.0087	0.0008 ± 0.0104	1.330	0.188
Left RN	0.0293 ± 0.0229	0.0247 ± 0.0249	0.903	0.370
Right RN	0.0785 ± 0.0280	0.0616 ± 0.0415	0.041	0.055
Left SN	0.0198 ± 0.0255	0.0067 ± 0.0320	1.832	0.072
Right SN	0.0341 ± 0.0391	0.0332 ± 0.0298	0.096	0.924
ACC	0.0279 ± 0.0322	0.0221 ± 0.0126	0.917	0.363
PCC	0.0179 ± 0.0174	0.0130 ± 0.0283	0.851	0.398

ACC, anterior cingulate cortex; CN, caudate nucleus; FWM, frontal white matter; GP, globus pallidus; HP, hippocampus; PC, parietal cortex; PCC, posterior cingulated; PU, putamen; RN, red nucleus; SN, substantia nigra; TH, thalamus.

*denotes *p* < 0.05.

The intraclass correlation coefficients were >0.90 for all the ROIs.

## 4 DISCUSSION

Insomnia is a widespread problem in the world. According to the literature, about 30% of adults experience occasional sleep difficulties, and 6%–13% meet the insomnia diagnostic criteria (Morin, LeBlanc, Daley, Gregoire, & Merette, 2006; Morin et al., 2011; Ohayon, 2002). In this study, the insomnia patients had poorer MMSE and MoCA scores compared with the normal controls. Cognitive impairments were frequent in people with insomnia. These findings are in agreement with the prior literature. Previously, many studies have compared the neuropsychological performance of individuals with insomnia and normal sleepers. Reliable cognitive differences between the insomnia patients and normal sleepers have been identified in many cognitive domains including working memory, episodic memory, and problem solving (Fortier‐Brochu, Beaulieu‐Bonneau, Ivers, & Morin, 2012).

Susceptibility‐weighted imaging is a new imaging technique that can measure the tissues iron concentration in vivo. Until now, the golden standard of brain iron concentrations is the autopsy examinations of 81 normal brains performed by Hallgren and Sourander (1958). In this study, a close correlation was found between regional phase shifts values of our healthy controls and the golden standard in the putamen, caudate nucleus, globus pallidus, substantia nigra, red nucleus, thalamus, frontal white matter, and hippocampus. These results indicated that our SWI method is effective and reliable.

In this study, compared with normal controls, the PI patients showed significant increased phase shift values in the left caudate nucleus, left putamen, left hippocampus, and bilateral thalamus. Close correlation was found between the phase shift value of the left hippocampus and MMSE scores. These results suggest that iron deposition in the left hippocampus plays an important role in the pathophysiological mechanism of cognitive impairment. The hippocampus is responsible for memory function including memory encoding and memory retrieval. Patients with PI commonly showed memory function deficits (Backhaus et al., 2006; Fulda & Schulz, 2001). Negative correlations of disturbed sleep and memory tasks performance have been reported (Fortier‐Brochu & Morin, 2014; Oosterman, van Someren, Vogels, Van Harten, & Scherder, 2009). Recently it has been found that insomnia patients show decreased hippocampal volume and atrophy in the cornu ammonis and dentate gyrus which associated with impaired cognitive functions (Joo, Kim, Suh, & Hong, 2014). In this study, we also found iron deposition in thalamus, caudate nucleus, and putamen of the PI patients. The thalamus plays a major role in arousal, awareness level, and activity regulation. It can control the sleep and wakefulness states (Steriade & Llinás, 1988). The caudate nucleus and putamen are parts of the basal ganglia. They are responsible for voluntary movement control and took part in many cognitive functions including memory, emotion, and learning (Bastos‐Leite et al., 2007; Kantarci et al., 2010). The precise mechanisms of the abnormal iron deposition in these brain regions of PI are not understood.

Using SWI technology, brain iron deposition has been reported in several other diseases previously. The iron concentrations of the substantia nigra in PD patients have been proved significantly increased and closely correlated with the UPDRS motor score (Zhang et al., 2010). Patients with AD demonstrated increased iron concentration in the temporoparietal, frontal, and parietal regions. High iron content has been proved to play an important role for formation of senile plaques in animal studies, which is the main pathologic manifestation of AD (McCrea, Harder, Martin, Buist, & Nichol, 2008; Nakada, Matsuzawa, Igarashi, Fujii, & Kwee, 2008). Several other diseases including multiple sclerosis, mild cognitive impairment, and amyotrophic lateral sclerosis were also proved associated closely with regional high deposition of iron (Chawla et al., 2016; Schweitzer et al., 2015).

In conclusion, in this study we found the PI patients have significant cognitive impairment and increased iron deposition in several brain regions. The iron concentration of the left hippocampus is a biomarker of cognitive impairment and may play an important role in the pathophysiological mechanism. This study had several limitations. Firstly, there is a relatively small sample size in this study. Secondly, as in previous literature, we considered that increasing susceptibility reflects increasing iron concentration. However, sometime several other metals such as manganese and copper may have the potential to cause susceptibility (Schenck & Zimmerman, 2004).
